# Methodological and Statistical Concerns Regarding “Can Sodium‐Glucose Co‐Transporter‐2 Inhibitors Improve Sleep Quality, Anxiety, and Quality of Life in Patients With Heart Failure?”

**DOI:** 10.1002/clc.70203

**Published:** 2025-09-01

**Authors:** Rajeev Gupta, Shekhar Vohra, Anshul Yadav, Neelesh Gupta, Rohit Mody

**Affiliations:** ^1^ Spectrum Medical Center, and Burjeel Royal Hospital Al Ain UAE; ^2^ Department of Cardiology, Mount Sinai Morningside Icahn School of Medicine at Mount Sinai New York USA; ^3^ Mahaveer Institute of Medical Sciences, and Research Bhopal India; ^4^ Central Cardiology Medical Center Bakersfield USA; ^5^ Mody Harvard Cardiac Institute & Research Centre‐ Krishna Super Specialty Hospital Bathinda India

**Keywords:** anxiety, heart failure, Pittsburgh Sleep Quality Index (PSQI), quality of life, SGLT‐2 inhibitors, sleep quality

## Disclosure

Use of AI for paraphrasing and in analyzing the statistical model used in the study.

## Conflicts of Interest

The authors declare no conflicts of interest.

Dear Editor,

The recent article by Erbay et al. reporting improved sleep quality, anxiety, and quality of life in heart failure patients receiving SGLT2 inhibitors [1] addresses a clinically important but underexplored area. However, several methodological limitations merit attention before these findings are generalized.

First, there was a significant baseline imbalance in Pittsburgh Sleep Quality Index (PSQI) scores between groups (5.0 vs. 6.0, *p* = 0.036). This difference, favoring the SGLT2 inhibitor group at baseline, may partially explain the magnitude of improvement observed. While within‐group analyses were performed, regression models should have included baseline PSQI as a covariate to mitigate this confounding [2].

Second, the multivariate logistic regression did not adjust for several potential confounders that could influence patient‐reported outcomes, including changes in diuretic dosing, concurrent initiation of other guideline‐directed medical therapies, and intercurrent hospitalizations. These variables are known to impact congestion, sleep, and mood in heart failure [3, 4].

Third, the subgroup analyses by ejection fraction status are based on small sample sizes, limiting statistical power and precision. Without reporting interaction *p*‐values, the assertion of consistent benefit across EF strata is premature [5].

Fourth, multiple SF‐36 domains and other secondary outcomes were tested without correction for multiplicity. In this setting, the risk of false‐positive findings is high, particularly in small observational cohorts [6].

Lastly, the study's observational, single‐center design precludes definitive causal inference, yet several statements in the discussion imply a treatment effect. This language should be tempered to reflect association rather than causation [7].

Given the increasing integration of SGLT2 inhibitors into heart failure care, it is critical that conclusions about novel patient‐reported benefits be supported by rigorous methodology. Randomized controlled trials incorporating objective sleep measures (e.g., polysomnography) and adequate adjustment for confounding are needed to validate these intriguing findings.

## Data Availability

This letter to the editor is a methodological and statistical critique of the published article “Can Sodium‐Glucose Co‐Transporter‐2 Inhibitors Improve Sleep Quality, Anxiety, and Quality of Life in Patients with Heart Failure?” and does not involve new patient enrollment, data collection, or analysis. No new datasets were generated or analyzed in this work. All information discussed is derived from the data presented in the original publication, which is publicly available through Clinical Cardiology.
